# Microbial Community Structure and Functions in Ethanol-Fed Sulfate Removal Bioreactors for Treatment of Mine Water

**DOI:** 10.3390/microorganisms5030061

**Published:** 2017-09-20

**Authors:** Malin Bomberg, Jarno Mäkinen, Marja Salo, Mona Arnold

**Affiliations:** VTT Technical Research Centre of Finland, P.O. Box 1000, FIN-02044 Espoo, Finland; jarno.makinen@vtt.fi (J.M.); marja.salo@vtt.fi (M.S.); mona.arnold@vtt.fi (M.A.)

**Keywords:** waste water treatment, archaea, fungi, high-throughput sequencing, PICRUSt, sulfate reducing bacteria, acetate, denitrification

## Abstract

Sulfate-rich mine water must be treated before it is released into natural water bodies. We tested ethanol as substrate in bioreactors designed for biological sulfate removal from mine water containing up to 9 g L^−1^ sulfate, using granular sludge from an industrial waste water treatment plant as inoculum. The pH, redox potential, and sulfate and sulfide concentrations were measured twice a week over a maximum of 171 days. The microbial communities in the bioreactors were characterized by qPCR and high throughput amplicon sequencing. The pH in the bioreactors fluctuated between 5.0 and 7.7 with the highest amount of up to 50% sulfate removed measured around pH 6. Dissimilatory sulfate reducing bacteria (SRB) constituted only between 1% and 15% of the bacterial communities. Predicted bacterial metagenomes indicated a high prevalence of assimilatory sulfate reduction proceeding to formation of l-cystein and acetate, assimilatory and dissimilatory nitrate reduction, denitrification, and oxidation of ethanol to acetaldehyde with further conversion to ethanolamine, but not to acetate. Despite efforts to maintain optimal conditions for biological sulfate reduction in the bioreactors, only a small part of the microorganisms were SRB. The microbial communities were highly diverse, containing bacteria, archaea, and fungi, all of which affected the overall microbial processes in the bioreactors. While it is important to monitor specific physicochemical parameters in bioreactors, molecular assessment of the microbial communities may serve as a tool to identify biological factors affecting bioreactor functions and to optimize physicochemical attributes for ideal bioreactor performance.

## 1. Introduction

Sulfate reducing bacteria (SRB) are widespread in anoxic environments and are used in diverse bioremediation and sulfate removal applications. Biological sulfate reduction (BSR) is the most widely applied method for sulfate removal from mine waters after gypsum precipitation [1]. The technology relies on anaerobic SRB, which use organic compounds or hydrogen gas as electron donors for the reduction of sulfate to hydrogen sulfide [2,3,4]. SRB can roughly be divided into two groups; those that oxidize organic compounds incompletely, producing acetate as their final product, and those that oxidize organic compounds completely all the way to CO_2_ [5]. Complete oxidizers are usually also able to utilize acetate as substrate, oxidizing it to CO_2_.

Suitable substrates (electron donors) that are economically sustainable, locally produced, or easily transported and stored, are key factors for the feasibility of treating mine water using a BSR bioreactor. Ethanol is a substrate that is fairly economic, may easily be transported to and stored at the mine site, and has successfully been applied before [6,7]. The first step in BSR when ethanol is used as electron donor produces acetate but also acidity (H^+^) (Equation (1); [4]). Acetate itself may also serve as substrate for SRB producing bicarbonate (Equation (2); [4]), which is subsequently oxidized to CO_2_ (Equation (3); [4]). Sulfate is simultaneously reduced to hydrogen sulfide (Equations (1) and (2)).

2CH_3_CH_2_OH + SO_4_^2−^ ⇋ 2CH_3_COO^−^ + HS^−^ + H^+^ + 2H_2_O, (1)

2CH_3_COO^−^ + 2SO_4_^2−^ ⇋ 4HCO_3_^−^ + 2HS^−^, (2)

4HCO_3_^−^ + H^+^ ⇋ CO_2_ + H_2_O, (3)

The complete oxidation of ethanol to CO_2_ increases the pH due to the conversion of bicarbonate to CO_2_ (Equation (3)).

Mine water often contains dissolved metals, which may need to be removed from the water before it is discharged into receiving water bodies. Metal removal occurs when these react with the hydrogen sulfide produced when sulfate is reduced, and precipitate as metal sulfides (Equations (4) and (5); [8]).

HS^−^ + 2Me^3+^ ⇋ 2Me^2+^ + S^0^ + H^+^, (4)

HS^−^ + Me^2+^ ⇋ MeS + H^+^, (5)

At low pH, metals may precipitate with sulfate as e.g., the iron-oxyhydroxysulfate schwertmannite at pH 2.5–4.5 or the iron-sulfate hydroxide jarosite at pH < 2.5 [9,10]. If metals are absent, the produced sulfide is either released or oxidized to S^0^ or even to sulfate [11].

Nagpal et al. [7] used ethanol as substrate for BSR in a fluidized bed reactor with as much as 95% of the inflowing sulfate removed with a hydraulic retention time (HRT) of 35 h (sulfate in ~2.5 g L^−1^, sulfate loading ~1.7 g L^−1^ d^−1^). The maximum sulfate removal rate of 6.33 g L^−1^ d^−1^ was measured with an HRT of 5.1 h. Nevertheless, in a similar experiment with a down-flow fluidized bed reactor, Celis et al. [6] acquired a sulfate removal efficiency of only 28% with a sulfate loading of 0.8–1.7 g L^−1^ d^−1^ and an HRT of 48 h. In both experiments acetate accumulation was detected in the effluent. In contrast to the study by Celis et al. [6], Nagpal et al. [7] reported that the sulfate removal rate was not affected by the accumulation of acetate. The different results obtained in these two studies may be due to differences in the microbial communities operating in the bioreactors. Sahinkaya et al. [4] reported the highest sulfate removal rates of 90% (feed sulfate concentration 2.5 g L^−1^) on ethanol with an HRT of 12 h.

Nevertheless, anoxic ethanol oxidation by bacteria proceeds by oxidizing ethanol to acetaldehyde and further to acetyl-CoA by the bifunctional alcohol dehydrogenase/aldehyde dehydrogenase enzyme (AdhE), or to acetaldehyde by alcohol dehydrogenase (ADH), whereafter the acetaldehyde is either oxidized by aldehyde dehydrogenase (ALDH) to acetate [12,13], or assimilated into biomass as ethanolamine. In addition, an increase in biomass could lead to an increase in the assimilatory sulfate reduction, where sulfate is reduced and taken up by the microorganisms as raw material for the synthesis of sulfur-containing amino acids, such as cysteine and methionine, which results in reduced amounts of free sulfide to be used for metal sulfide precipitation.

Compounds such as nitrate and nitrite may also influence the sulfate reduction and sulfate removal processes. For example, nitrate reducing and sulfide oxidizing bacteria may re-oxidize the sulfide back to sulfate [14]. Nitrite produced in the nitrate reducing process has an inhibitory effect on sulfate reducing bacteria with concentrations as low as 5 mM nitrite either completely or partly inhibiting the sulfate reduction [15]. This inhibition may be overcome if the sulfate reducer contains the nitrite reductase (Nrf) for converting nitrite to ammonia [15]. In fact, nitrate and nitrite are used to prevent sulfide formation or to remove produced sulfide in e.g., sewage systems, oil production operations, and other anaerobic sulfate-rich environments [16].

In this study, we hypothesized that ethanol is a suitable electron donor for the biological sulfate reduction in the treatment of sulfate-rich water originating from a northern mine. Based on previous tests in our laboratory, we also hypothesized that the microbial community in our sulfate removal bioreactors had high microbial diversity, but that the communities were dominated by sulfate reducers. We aimed to clarify the composition of the microbial communities operating in the bioreactors, when ethanol was used as electron donor, using modern high throughput molecular methods, and monitor the physicochemical condition in the reactors over time in relation to sulfate removal from the mine water. Lastly, we aimed to obtain a comprehension of the possible metabolic processes the microbial communities of the bioreactors could be capable of by studying predicted metagenomes of the microbial communities produced with the PICRUSt software [17] in order to allow for modifications in how the bioreactors are run in the future.

## 2. Materials and Methods

### 2.1. Bioreactors

The experimental setup consisted of three anaerobic 0.7 L column reactors. Reactor 1 was operated as an upflow anaerobic sludge blanket reactor (UASB), while Reactor 2 and Reactor 3 were operated as fluidized bed reactors (FBR). Reactor 2 and Reactor 3 were operated with 10% fluidization volume with crushed expanded clay particles (Filtralite NC 0.8–1.6) or activated carbon as carrier materials, respectively. Later, carrier material for Reactor 2 was changed to 0.5–1.0 mm particle-sized sand, because Filtralite (0.8–1.6 mm) clogged the pipelines. The volume of the sludge blanket and carrier materials was 0.3 L, which was used as the effective volume of the reactor and basis for e.g., HRT calculations.

The microbial inoculum for all reactors was anaerobic, sulfide-producing granular sludge from a Finnish operating industrial plant treating ethanol-containing wastewater. Ethanol was used as substrate, due to its common utilization in commercial applications, but also due to its easy storage and transportation to remote mine sites. Water from a neutralization pond from an operating mine in Northern Finland was used in the reactors, with an original pH of 6.4. The reactor experiment was started by adding the mine water to the reactors, followed by a 30 min N_2_ flushing to remove oxygen. A 300 mL inoculum of anaerobic granular sludge was added to Reactor 1. For the FBR Reactors 2 and 3, carrier materials were first added, whereafter the fluidization was fixed and an inoculum of 60 mL anaerobic granular sludge was added to the reactors. The HRT in the experiment was 173 h, during both the ramp up and actual water treatment phase.

The chemical composition (33 elements) of the mine water was examined at the Finnish Accreditation Services (FINAS) accredited analysis laboratories (T025, EN ISO/IEC 17025) Labtium Oy (Espoo, Finland) and MetropoliLab (Espoo, Finland) (Table 1).

The feed solution for the reactors was a mixture of mine water, nutrients, and ethanol as substrate, resulting in 8.5–9.0 g L^−1^ SO_4_, 56 mg L^−1^ KH_2_PO_4_, 137 mg L^−1^ (NH_4_)_2_HPO_4_, 11 mg L^−1^ ascorbic acid and 11 mg L^−1^ yeast extract. The ethanol dosage was calculated from the chemical oxygen demand (COD) according to the estimate that one gram of sulfate requires 2 g of COD and the COD of ethanol is 1440 g L^−1^. During the ramp-up phase and the first 27 days of the experiment the ethanol dosage was kept at 15% of required COD in order to prevent acetate formation and accumulation in the reactors. From day 28 to day 104 the ethanol dose was increased to 160% of the required COD in order to secure sufficient substrate concentration for efficient sulfate removal. During days 105–171 the sulfate concentration was decreased to 3.0 g L^−1^ by dilution with distilled water and the ethanol dose was decreased to 120% of the calculated required concentration for full sulfate removal. The feed solution was kept in a plastic container at room temperature by the bioreactors throughout the experiment. pH monitoring showed that the pH fluctuated only slightly, between 6.4 and 7.4 over the time of the experiment.

The reactors were monitored for pH, RedOx potential, and sulfate and sulfide concentrations twice per week from effluent collected directly from the bioreactors. pH and RedOx potentials were measured with a Consort multi-parameter analyser C3040 equipped with Van London-pHoenix Co. electrodes (Ag/AgCl in 3 M KCl). To prevent sulfide loss, sample collection bottles were filled to the top and sealed with air-tight caps until measurements. In addition, the RedOx measurement was performed within 1 min of the sampling, followed by sulfide measurements using the Hach Lange LCK653 kit within 2 min of the sampling, and lastly, pH and sulfate concentrations were measured using a pH meter and the Hach Lange LCK353 kit, respectively. On day 88 and 91, SO_4_, ethanol, acetate, and nutrients were measured by a FINAS accredited external laboratory.

### 2.2. Microbiology

The microbiology of the inoculum and reactors (Day 101 and 140) was determined using DNA-based high throughput (HTP) sequencing techniques. Microbial DNA was extracted from 0.5 g inoculum sludge and from 2 mL samples of effluent from the bioreactors. Excess liquid was first removed from the solids by centrifugation for 10 min at 13,000 rpm in a table-top centrifuge (Eppendorf, Hamburg, Germany), whereafter the supernatant was carefully decanted. The DNA extraction was performed with the NucleoSpin Soil DNA extraction kit (Macherey-Nagel, Düren, Germany) and proceeded according to the manufacturer’s instructions using the SL1 lysis buffer and Enhancer solution. The DNA was eluted in 100 μL elution buffer. 

The total number of bacteria and SRB in the inoculum and reactors was determined by quantitative PCR (qPCR) targeting the bacterial 16S rRNA genes and the *dsr*B genes of the SRB. The bacteria were targeted by amplifying an approximately 200 bp fragment of the 16S rRNA gene using primers P1 and P2 [18] and the *dsr*B genes by using the primers *dsr*2060F and *dsr*4R [19,20] as previously described [21,22].

The microbial community composition in the inoculum and bioreactor effluents was determined by characterizing the whole community profiles targeting the 16S rRNA genes of the bacteria and archaea using primer pair S-D-Bact-0341-b-S-17/S-D-Bact-0785-a-A-21 for bacteria and primer pair S-D-Arch-0349-a-S-17/S-D-Arch-0787-a-A-20 for archaea [23] targeting the v3 and v4 regions of the gene, and the ITS region for the fungi using primers ITS1 and ITS2 [24]. PCR amplification was performed in parallel 25 μL reactions for every sample containing 1× MyTaq^TM^ Red Mix (Bioline, London, UK), 20 pmol of each primer, up to 25 μL molecular-biology-grade water (Sigma-Aldrich, Munich, Germany) and 2 μL of DNA. The PCR program consisted of an initial denaturation step at 95 °C for 3 min, 35 and 40 cycles of 15 s at 95 °C, 15 s at 50 °C, and 15 s at 72 °C, for bacteria and archaea, respectively, and a final elongation step of 30 s was performed at 72 °C. The PCR products were verified with agarose gel electrophoresis. Amplicons were sent to Ion Torrent sequencing on the PGM platform (Bioser, Oulu, Finland) and amplicons were purified before sequencing at Bioser.

The sequence reads obtained from Ion Torrent sequencing were subjected to quality control using the QIIME software version 1.9 [25] using a minimum quality score of 20, minimum and maximum sequence length of 200 bp and 600 bp, respectively, maximum primer mismatch of 2 nucleotides (nt) and maximum homopolymer stretches of 8 nt. Adapters, barcodes, and primers were removed from the sequence reads, and chimeric sequence reads were removed from the data set with the USEARCH algorithm [26] by de novo detection and through similarity searches against the Greengenes reference dataset (version gg_13_8) [27] with bacterial and archaeal sequences, and UNITE reference dataset (version sh_taxonomy_qiime_ver7_97_s_31.01.2016) [28] with fungal sequences.

The sequences were grouped into Operational Taxonomic Units (OTUs), following the open-reference OTU-picking protocol of QIIME, using UCLUST [26] to cluster sequence reads at 97% sequence similarity. Taxonomiy from the domain- to species-level was assigned to OTUs via representative OTU sequences with the RDP classifier algorithm at minimum confidence threshold of 80% [29] for bacterial and archaeal sequences. Taxonomic assignments for the fungal ITS sequences were made using the BLAST algorithm with a maximum E-value of 0.001 [30]. Sequence reads obtaining no taxonomical assignments in the analyses were excluded from the datasets.

Microbial metabolic pathways were estimated using the PICRUSt software, version 1.0.0 [17]. The principle of the PICRUSt analysis is thoroughly described by the developers [17], and will only shortly be explained here for clarity. PICRUSt employs the ancestral state reconstruction (ASR) method to predict the gene content of uncultured microorganisms, for which a genome is not yet available. Despite genome plasticity due to gene loss, duplication, or transfer, the assumption is that the gene content between closely related taxa is more similar than between distantly related taxa. Nevertheless, uncertainties arise because microbial genomes may change rapidly over evolutionary time, a fact that should be considered when interpreting these predictions, and the prediction are accompanied by a 95% confidence interval. PICRUSt links 16S rRNA gene sequences of complete reference genomes and genomes identified from uncultured bacteria and archaea (environmental samples) to the annotated genomes. The 16S rRNA gene sequences of the reference genomes are contained in a phylogenetic tree and linked to the functional annotations (e.g., KEGG orthologs) of the gene content of the reference genomes. This analysis is based on 16S rRNA gene data that has been analyzed using the closed OTU picking method in QIIME using the Greengenes reference dataset version gg_13_5 database [27] for taxonomic assignments. The number of taxa present in the samples was normalized by predicting the number of 16S rRNA gene copies of each identified taxon using PICRUSt, i.e., the resulting OTU table corresponds to the relative number of microorganisms, not 16S rRNA genes. The metagenome was then predicted for the normalized data using PICRUSt’s pre-calculated KEGG ortholog database files, which estimates the number of gene copies of each gene family per microorganism. The most common bacterial OTUs responsible for specific enzymes or metabolic processes were identified using the metagenome_contribution.py command in PICRUSt [17] and the abundances of these bacterial groups were plotted separately for each function in R [31].

The Nearest Sequenced Taxon Index (NST) for evaluating the novelty of the organisms included in an OTU table (described in [17]) with respect to previously sequenced genomes was calculated for each sample. The NSTI is the sum of branch lengths between an OTU in the Greengenes tree to the nearest tip in the tree with a sequenced genome weighted by the relative abundance of that OTU. All OTU scores are then summed to give a single NSTI value per microbial community sample.

The sequence data has been submitted to the European Nucleotides Archives (ENA, http://www.ebi.ac.uk/ena) under study number PRJEB21687 (Accession number ERS1812840-ERS1812860).

The viability of the microorganisms in the reactors was examined using the LIVE/DEAD^®^ *Bac*Light™ Bacterial Viability (L/D) (Thermo Fisher Scientific, Waltham, MA, USA) staining kit as recommended by the manufacturer. Bioreactor effluent samples of 5 mL were obtained at the end of the experiment and were stained with the L/D reagents for 30 min after which the microbial cells were concentrated on black 0.2 μm pore-size polycarbonate membrane filters (Isopore™ Membrane filters, 0.2 μm GTBP, Millipore, MA, USA) with a Millipore 1225 Sampling Manifold (Millipore, MA, USA) using low vacuum suction. The filters were mounted on microscopy slides, covered with a cover glass, and examined under UV light with an epifluorescence microscope (Olympus BX60, Olympus Optical Ltd., Tokyo, Japan) at 100× magnification.

### 2.3. Statistical Analyses

The Pearson correlation between different measured parameters (pH, sulfate removal) was statistically tested using PAST [32] on the monitoring data collected until day 101. After day 101 the sulfate load was changed. The bioreactors were tested separately or combined. Only parameters for which normal distribution was confirmed by all three tests in PAST (Shapiro–Wilk, Anderson–Darling and Jarque–Bera (both p (normal) and p (Monte Carlo)) were further analyzed. The Bonferroni correction model for identification of false positives was used. 

## 3. Results

### 3.1. Performance of the Bioreactors and Chemical Aspects

**Figure 1:**
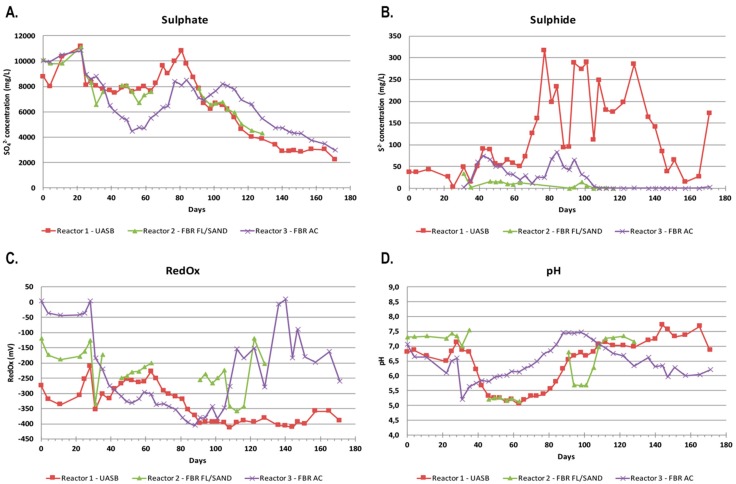
Parameters measured in the effluent of the bioreactors over time: (**A**) the concentration of sulfate (mg L^−1^); (**B**) the concentration of sulfide (mg L^−1^); (**C**) the redox potential (mV); and (**D**) the pH over the span of the experiment. The red, green, and purple curves correspond to Reactor 1, Reactor 2, and Reactor 3, respectively.

The experiment started (day 0) after the ramp up phase when all bioreactors had reached negative RedOx-potential values. During the first 20 days, with the calculated dosage of ethanol for full sulfate removal kept on 15%, the sulfate levels increased above the concentration of the influent water, after which the concentration of sulfate rapidly decreased in all reactors (Figure 1). In Reactors 1 and 2, the sulfate concentration decreased from the original 8500–9000 mg L^−1^ to approximately 8000 mg L^−1^, and the sulfate removal rate remained at approximately 10–20% and 100–250 g/m^3^ d, respectively. In Reactor 3, the decrease was stronger, resulting in a sulfate removal rate of approximately 45% and 500 g/m^3^ d, respectively. The decrease in the concentration of sulfate in the reactors started right before the ethanol dosage was increased to 160%. After day 60, the sulfate concentration started to increase strongly in all reactors, with the sulfate concentration in Reactor 1 increasing above that of the influent sulfate concentration. During this period, Reactor 2 was damaged due to smaller particles of the Filtralite material, resulting in wearing of the hoses. Filtralite was changed to sand, and the reactor was inoculated with 200 mL of effluent from reactor 3. Due to the fluctuating sulfate removal rate, the concentration of both sulfate and ethanol in the feed was decreased on day 105. Despite this modification, the sulfate removal after day 105 remained low.

When the ethanol dosage was increased to 160% (day 28), the pH of the reactors started to decrease. For Reactor 1 the decrease was remarkable, from pH 7.1 to 6.2 between days 28–39. On day 39, the acetate concentration in Reactor 1 was 6.8 g L^−1^, indicating that acetate was accumulating in the system causing a decrease in the pH level (Equations (1) and (2)). The pH continued to decrease and therefore NaHCO_3_ was added to Reactor 1 between days 81–88 (a total of 3 g) to raise the pH. Simultaneously, the acetate concentration was 2.2–3.4 g L^−1^ (Table 2). In Reactor 3, the pH decrease was not so dramatic and increased without NaHCO_3_ to pH > 7 on day 84, although the concentration of acetate was approximately 100 mg L^−1^.

Sulfide formation and sulfate removal in the reactors started on day 30 (Figure 1). After day 70, the sulfide concentration in Reactor 1 was rapidly increasing and fluctuating between 100–300 mg L^−1^, whereas in Reactor 3 no similar increase was observed and the sulfide concentration was less than 100 mg L^−1^. Thus, the production of sulfide did not correlate with the amount of sulfate removed as measured from the effluent.

**Figure 2:**
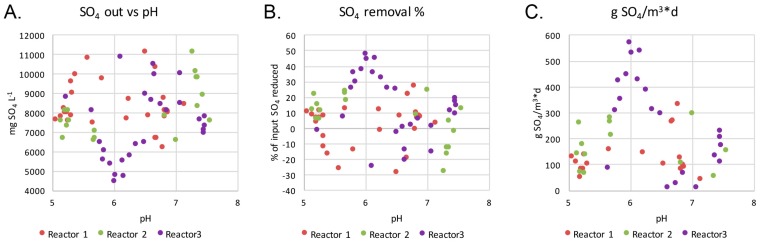
(**A**) The concentration of sulfate (mg L^−1^), (**B**) the percent of input sulfate removed and (**C**) the total amount of sulfate (g) removed per m^3^ of treated water per day plotted against the measured pH of the efflux of the reactors.

The highest sulfate removal rate was achieved in Reactor 3 (Figure 2). The highest level of sulfate removal correlated with a pH value of 5.5 to 6.5 and the highest amounts of removed sulfate per day correlated negatively and significantly with the effluent pH (−0.58, *p* < 0.01). No similar correlations were observed in the other reactors.

### 3.2. Microbiology

**Figure 3:**
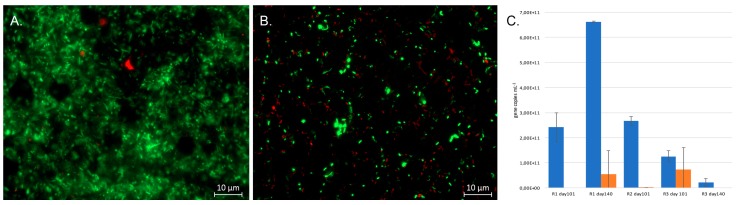
Live/Dead images of the effluents of Reactors 1 (**A**) and 3 (**B**) at the end of the experiment. Green indicates live cells and red are dead cells. The size marker is 10 μm. (**C**) The concentration of bacterial 16S rRNA gene copies (blue) and *dsr*B gene copies (orange) in the effluents (mL^−1^) estimated by qPCR. The error bars show standard deviation and each bar represents the average value of 3 replicate qPCR reactions.

The number of bacterial 16S rRNA gene copies mL^−1^ effluent exceeded 10^11^ copies mL^−1^, with the exception of Reactor 3 day 140 (Figure 3). The number of *dsr*B genes mL^−1^ was generally lower than that of the bacteria, ranging from 0.3% of the amount of 16S rRNA genes in Reactor 2 on day 101 but reaching up to 58% of the 16S rRNA gene concentration in Reactor 2 on day 101. The microbial communities in the reactors consisted of live microorganisms, as indicated by the Live/Dead microscopy of Reactor 1 and 3 at the end of the experiment (Figure 3).

**Figure 4:**
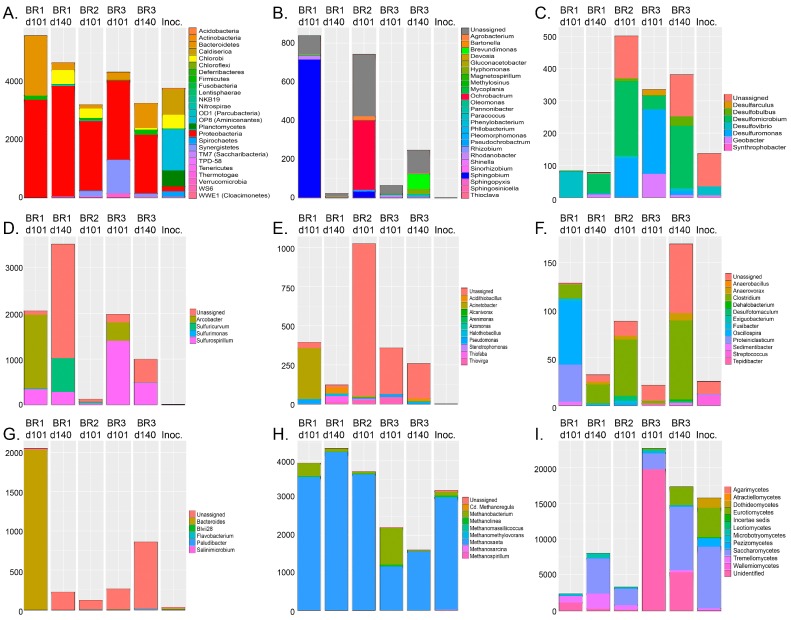
The microbial community profiles identified by high throughput (HTP) sequencing of the bioreactors (BR) and the inoculum (Inoc.): (**A**) The abundances of different bacterial phyla; (**B**) alphaproteobacterial; (**C**) deltaproteobacterial; (**D**) epsilonproteobacterial; (**E**) gammaproteobacterial; (**F**) Firmicutes; (**G**) Bacteroidetes genera; (**H**) archaeal genera; and (**I**) fungal classes. In all figures, the sample order from the left is Reactor 1 at day 101 and day 140, Reactor 2 at day 101, Reactor 3 at day 101 and 140, and the inoculum, as indicated in (**A**). The Y-axis shows the number of sequence reads. The taxonomic groups are shown by the color charts in each figure.

The bacterial groups expressing the highest relative abundance in the original inoculum were heterotrophic and sulfate assimilating Aminicenantes (former OP8, 49%) and thiosulfate-reducing Caldiserica (Figure 4), respectively. In the reactors, the relative abundances of bacterial groups in the communities changed to contain mostly proteobacteria (56–80%, Figure 4B–E) and Bacteroidetes (4–32%, Figure 4G). In Reactors 1 and 3 the Proteobacteria belonged to sulfur-oxidizing *Sulfuricurvum*, *Sulfurospirillum*, *Thiobacillus*, *Thiofaba*, and *Thiomonas*, in total 24% and 80% of the bacterial community (Figure 4D). In Reactor 2 (day 101), the most abundant bacteria belonged to undetermined gammaproteobacterial clades (25%, Figure 4E) and the alphaproteobacterial *Ochrobactrum* (20%, Figure 4B), which is also known to use nitrite reduction to power anaerobic oxidation of reduced sulfur species [33]. The SRB detected in the reactors belonged to the deltaproteobacterial genera *Desulfobulbus*, *Desulfomicrobium*, *Desulfovibrio*, and *Desulfuromonas* and contributed between 1 and 15% of the bacterial community (Figure 4C). Small amounts of Firmicutes SRB belonging to the *Desulfotomaculum* genus were detected in Reactor 2 after 101 (0.2%) and in Reactor 3 after 140 days of operation (0.5%, Figure 4F).

The archaeal communities in the inoculum and in all reactors consisted mostly of members of the methanogenic genus *Methanosaeta*, which use acetate for methane production (Figure 4H). Nevertheless, in Reactor 3 after 101 days of operation, almost half of the detected archaea belonged to CO_2_ and H_2_ utilizing *Methanobacterium*, which otherwise were present at only low abundance in all effluent samples.

Most of the fungi detected in the inoculum and reactors belonged to different families of Ascomycota (51–97%) (Figure 4I). However, Reactor 1 and 2 also contained a considerable amount of Basidiomycota (26–47%). The most common fungi were the Saccharomycetes and Eurotiomycetes, which both use fermentation as a mean for energy production [34]. In addition, Reactor 3 had a high abundance of unidentified fungal groups.

**Figure 5:**
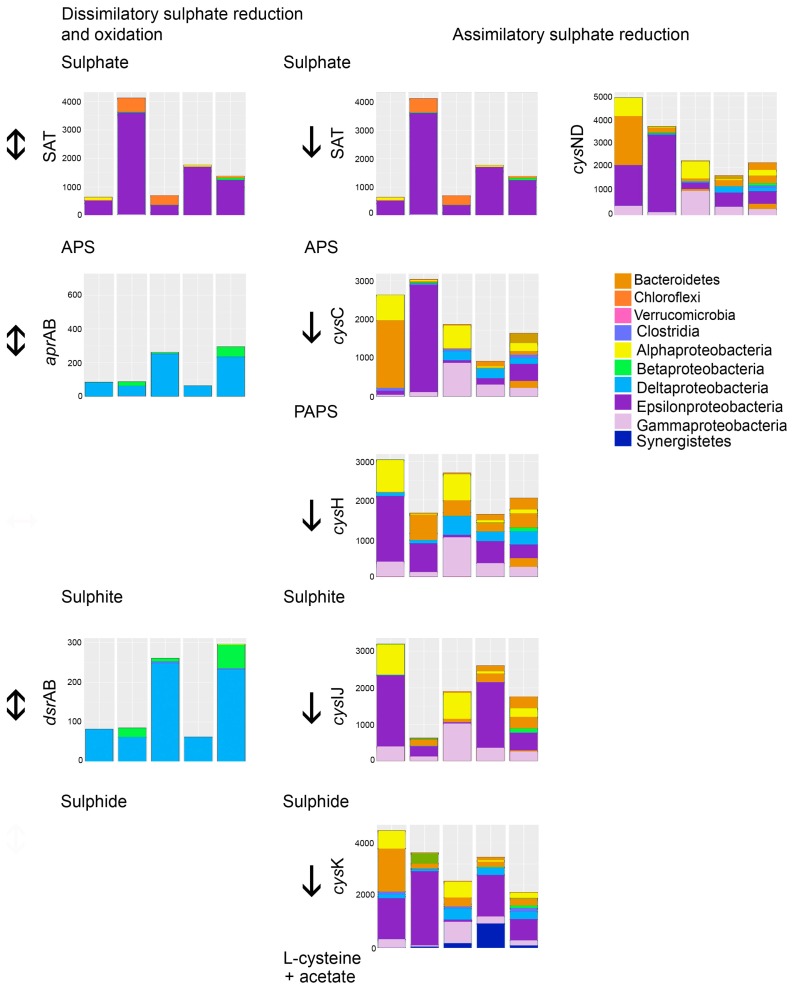
The bacterial groups responsible for the different steps of biological sulfate reduction processes in the bioreactors. Acetate is produced in the reaction where l-cysteine is produced from hydrogen sulfide + Pyruvate + ammonia. The *Y*-axis shows the number of sequence reads detected. The taxonomic groups are shown by the color charts in each figure. SAT—sulfate adenylyltransferase, *apr*AB—adenylylsulfate reductase, subunit A and B, *dsr*AB—dissimilatory sulphite reductase, subunit A and B, *cys*ND—sulfate adenylyltransferase subunit N and D, *cys*C—adenylylsulfate kinase, *cys*H—phosphoadenosine phosphosulfate reductase, *cys*IJ—assimilatory sulphite reductase (NADPH dependent), *cys*K—cysteine synthase A. The columns in each chart from the left; BR1 day 101, BR1 day 140, BR2 day 101, BR3 day 101, BR3 day 140.

The Nearest Sequenced Taxon Index (NSTI) value of the PICRUSt predicted metagenomes was on average 0.91 (±0.01 standard deviation) per sample. The estimated metagenome analyses indicated a several orders of magnitude higher relative abundance of bacterial groups able to perform assimilative sulfate reduction in comparison with dissimilatory sulfate reducing bacteria (Figure 5). The main SRB (dissimilatory sulfate reduction) belonged to deltaptoreobacteria, which also were predicted to contain the genes for the whole enzyme chain needed for dissimilatory sulfate reduction (Figure 5). However, the assimilatory sulfate reduction chain to sulfide was predicted to be present in the epsilon-, alpha- and gammaproteobacteria, Bacteroidetes, and Chloroflexi as well as in the Deltaproteobacteria. Interestingly, genes for the synthesis of the amino acid l-cysteine from sulfide, pyruvate, and ammonia were predicted to be especially abundant in a wide variety of bacterial groups. In this process acetate is released as a side product.

Genes needed for the SOX system, for the oxidation of thiosulfate to sulfate, were not detected in the predicted metagenomes.

**Figure 6:**
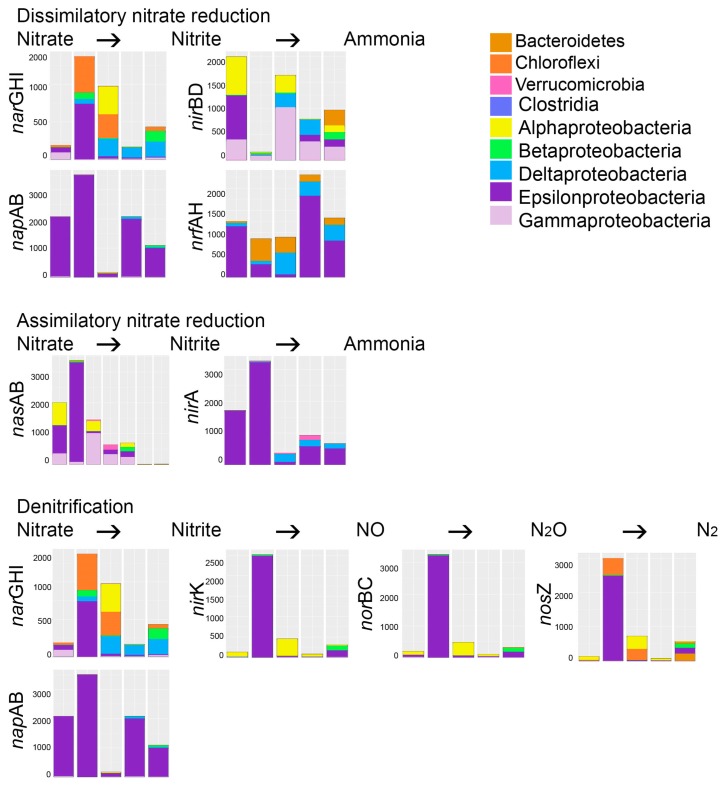
The bacterial groups responsible for the different steps of denitrification and nitrification processes in the bioreactors. The Y-axis shows the number of sequence reads. The taxonomic groups are shown by the color charts in each figure. *nar*GHI—nitrate reductase, *nap*AB—periplasmic nitrate reductase, *nir*BD—nitrite reductase (NADH dependent), *nrf*AH—nitrite reductase (cytochrome c-552), *nas*AB—assimilatory nitrate reductase, *nir*A—ferredoxin-nitrite reductase, *nir*K—nitrite reductase (NO-forming), *nor*BC—nitric oxide reductase, *nos*Z—nitrous-oxide reductase. The columns in each chart from the left; BR1 day 101, BR1 day 140, BR2 day 101, BR3 day 101, BR3 day 140.

Genes for both dissimilatory and assimilatory nitrate reduction via nitrite to ammonia were predicted in all effluent samples (Figure 6). The *nap*AB and *nrf*AH genes for dissimilatory nitrate reduction were predicted to be especially common among the epsilonproteobacteria, while the *nar*GHI and *nir*BD genes were predicted to be present in more diverse bacteria. Assimilatory nitrate reduction was mainly predicted among the epsilonproteobacteria (*nas*AB, *nir*A) and the gammaproteobacteria (*nir*A). Genes for the denitrification pathway (from nitrate > nitrite > NO > N_2_O > N_2_) to N_2_ were common among the epsilonproteobacteria, but mainly only in Reactor 1 on day 140. Genes for nitrification (i.e., oxidation of ammonia to nitrite) or anammox (anaerobic nitrite reduction to hydrazine, or ammonia oxidation to hydrazine and further to N_2_) were not predicted in any of the effluents.

**Figure 7:**
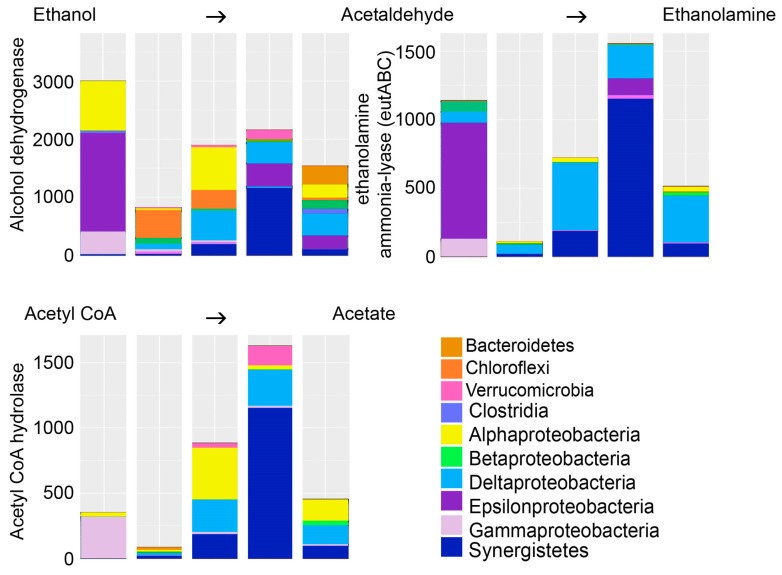
The bacterial groups able to oxidize ethanol into acetaldehyde and further into ethanolamine, which serves as precursor for the synthesis of amino and fatty acids. Acetate is produced through fermentation by oxidation of pyruvate to acetate via Acetyl CoA. The *Y*-axis shows the number of sequence reads. The taxonomic groups are shown by the color charts in each figure. The columns in each chart from the left; BR1 day 101, BR1 day 140, BR2 day 101, BR3 day 101, BR3 day 140.

Ethanol was tested in this study to serve as electron donor for biological sulfate removal from mine wastewater. In biological processes, ethanol is converted to acetaldehyde by the alcohol dehydrogenase (Figure 7). However, the enzyme aldehyde dehydrogenase that turns acetaldehyde to acetate was not predicted. Instead, the bacterial predicted metagenomes contained relatively high abundances of ethanolamine ammonia-lyase, which produces ethanolamine from acetaldehyde and ammonia. Ethanolamine, in turn, is used by many microbial groups as a carbon and nitrogen source or storage [35] and is a major component in cell membranes [36].

## 4. Discussion

Biological sulfate reduction as a technique to clean mine water for reuse or discharge, as well as a method for capturing of metals as metal sulfides, is a desirable method because it could ultimately be both energy- and cost-efficient as well as a low-labour method to be applied on the mine premises. Some specific environmental parameters are required in order for SRB to perform sulfate reduction, such as low redox potential and suitable electron donors. Mine waters commonly contain only small amounts of organic matter and an external carbon source and electron donor need to be provided for biological sulfate reduction. Numerous options for substrates are available, and ethanol is one of the commonly used ones, mainly for its relatively low cost, easy transportation, and good suitability for a wide range of SRB. Ethanol has been used with variable results in previous studies (e.g., [6,7]). Biological ethanol oxidation is catalyzed by the alcohol dehydrogenase enzyme to produce acetaldehyde in an energy-demanding and acidity producing reaction. Acetaldehyde in turn is either further oxidized to acetic acid and H^+^ in an energy gaining reaction by the aldehyde dehydrogenase enzyme, or can be condensed with ammonia by the enzyme ethanolamine ammonia-lyase to form ethanolamine. Ethanolamine is a major constituent of bacterial membranes and functions also as carbon and nitrogen storage to be used by the bacteria during starvation.

In our study, we detected accumulation of acetate in the effluent, leading to acidification of the bioreactors and a decrease in the sulfate removal process (Table 2). Celis et al. [6] reported similar results showing that the sulfate reduction process was hampered as acetate accumulated. Nevertheless, Napgal et al. [7] reported accumulation of acetate as high as 1.5–2.7 g L^−1^ without detecting any inhibition in the sulfate removal rate. As mentioned above, acetate may be produced through the oxidation of ethanol via acetaldehyde, and if not further oxidized to acetyl CoA by the acetyl-CoA synthetase enzyme, acetate accumulates in the solution. We used the PICRUSt software [17] in order to predict the bacterial metagenomics content in the bioreactors. PICRUSt uses the 16S rRNA genes of known reference genomes as base for the prediction of the genomic content of uncultured microbial taxa closely related to reference microorganisms. This tool provides an estimation functional potential of an uncultured community. Although changes in microbial genomes based on loss or gain of genes cannot be determined using PICRUSt, it may be assumed that closely related taxa identified by their 16S rRNA gene may share a higher degree of features than distantly related taxa. With these uncertainties in mind, we found that our predicted metagenomes contained a wide variety of bacteria that were estimated to have the alcohol dehydrogenase enzyme needed for the oxidation of ethanol to acetaldehyde. In contrast, the aldehyde dehydrogenase enzyme for the oxidation of acetaldehyde to acetate was not predicted. Instead, the acetaldehyde was predicted to be used for the production of ethanolamine (Figure 7). We found no evidence for the presence of acetyl-CoA synthetase in the predicted metagenomes.

The predicted metagenomes indicated that acetate could be produced through fermentation processes, where pyruvate produced in the glycolysis is fermented to acetyl CoA and further to acetate (Figure 7). Another source of acetate is the assimilatory sulfate reduction pathway, which was predicted to be common in the bacterial communities (Figure 5). SRB using dissimilatory sulfate reduction as a source of energy formed a surprisingly small part of the microbial communities detected in the reactors (Figure 2 and Figure 6). Instead, the great majority of the bacteria detected were groups that conduct assimilatory sulfate reduction, i.e., reduce sulfate to be used as building material for the production of e.g., sulfur-containing amino acids, such as l-cysteine (Figure 5). In the production of l-cysteine, acetate is released, which may be a likely cause for the accumulation of acetate in the effluent. In this case, the formation of sulfide would be non-existent. The abiotic removal of sulfate from the bioreactor solution in the form of metal-oxyhydroxysulfates or metal-sulfate hydroxides can also be excluded due to the lack of any higher concentrations of iron or aluminium to serve as the metal ions, and a generally too high pH for these precipitates to form [9,10].

Ammonia was added as nitrogen source for the microbial consortia and ammonium-N was also present in the original mine water, but no evidence for ammonia oxidation was detected in the predicted bacterial metagenomes. However, the ability to form ammonia through both dissimilatory and assimilatory nitrate reduction was predicted in all bioreactors. Nitrate was not found in high amounts in the original mine water (Table 1), but nitrite was abundantly available. Nitrate has been shown to completely or partly inhibit the sulfate reduction of strains of *Desulfovibrio* spp. at a concentration as low as 5 mM [15]. In comparison, the molar concentration of nitrite in the mine water used in our study was 78 mM, which may strongly affect the SRB. Nitrite could either be directly reduced to ammonia in the assimilatory and dissimilatory nitrate reduction pathways (Figure 6), or more likely, the nitrite in the mine water oxidized to nitrate during storage of the mine water and nitrate was used as electron acceptor for oxidation processes. In this case, the nitrate could be used as an electron acceptor for the oxidation of sulfide by sulfide-oxidizing bacteria [15].

The initial concentration of sulfate has been shown to affect the efficiency of the biologic sulfate reduction with optimal sulfate reduction observed at concentrations of approximately 2.5 g L^−1^ sulfate [4,37]. At higher concentrations, the reduction rates decrease and the risk for acetate accumulation increases, which in turn may further inhibit the sulfate reduction process [4]. In our case, the sulfate concentration of the mine water over the first 104 days was 8.5–9.0 g L^−1^, which is almost 4 times higher than reported for optimal growth of SRB [4,37]. Thus, a combination of too high concentration of sulfate with ethanol as electron donor in addition to long HRT could have resulted in the accumulation of acetogenic and non-SRB biomass.

SRB need anoxic conditions with negative redox potential of at least −150 to −200 mV for their metabolism to function properly [38]. If the redox potential is higher than this, for example in the presence of oxygen, sulfate remains stable and no sulfide is produced [39]. Sulfate reduction usually works best at pH 7–8 [2,40], although there have been experiences with comparable sulfate reduction even in very acidic (pH 4) environments [41]. In our bioreactors, the pH fluctuated between as low as 5 to above 7.5, but the highest removal was seen at pH 6 in Reactor 3, where up to 48% of the input sulfate was removed. This occurred during day 39–73, with the peak on days 52–59. The redox in Reactor 3 during this period was −300 mV and below. Both Reactor 1 and Reactor 3 reached even more negative redox values of below −400 mV although the highly negative redox did not increase the sulfate removal.

Excess sulfate can also affect the reactor performance, as it may result in elevated redox potential and lowered pH and thus diminish the sulfate reduction activity [42]. These conditions may also favour other microbes over SRB in the reactors [43]. This is in agreement with our results, as only 2–15% of the bacterial communities consisted of SRB. The presence of high amounts of acetate-utilizing methanogens and the relatively low abundance of SRB is consistent with the low sulfate reduction activity generally observed in the reactors. In addition, the high abundance of bacteria assimilating sulfate into biomass (e.g., l-cysteine) caused acetate to accumulate. It is possible that the long HRT (173 h) allowed non-SRB microorganisms to accumulate and form biomass that competed with the SRB.

The presence of aceticlastic methanogens in the bioreactors can be explained by the prevalent acetate produced in the bioreactors. Nevertheless, Dar et al. [43] and Kristjansson & Schönheit [44] argued that methanogens and acetogens are generally outcompeted by sulfate reducers when suitable electron acceptors and adequate sulfate are available, due to the SRB’s higher affinity for H_2_. However, Ozuolmez et al. [45] showed that *Desulfovibrio vulgaris* and *Methanosaeta concilii* can coexist in cultures, where H_2_ leaking from the aceticlastic methanogenesis of *M. concilii* is used for sulfate reduction by *D. vibrio*. In addition, the authors showed that even the acetotrophic SRB *Desulfobacter latus* and *M. concilii* coexisted in the cultures rather than competing for resources.

The fungi present in the bioreactors may also add to the production of acetate and overall acidity, because of fermentation. Fungi are able to utilize many different types of organic compounds as substrate, but ultimately, in the absence of oxygen, the Saccharomycetes and Eurotiomycetes can turn to fermentation, in which they produce acetate or other organic acids and H^+^ [29].

Despite the easy accessibility of ethanol as electron donor for biological sulfate removal processes, the ethanol in our case was incompletely oxidized. In addition, the reactor conditions may have supported more biomass growth and development of a strong community of fermenters. It is possible that more efficient biological sulfate reduction and thus sulfate removal could be achieved with shorter HRT, which would allow for the SRB to reduce the influx sulfate without allowing for excess biomass growth, and to screen for microbial consortia that are able to oxidize acetaldehyde to acetyl-CoA and further to CO_2_ via acetate. It could also be possible to employ genetically modified microbial species or consortia in order to design bioreactor consortia to perform specific processes for optimal results and better control of the bioreactor processes.

## 5. Conclusions

Based on previous tests on our laboratory, we hypothesized that the microbial community in our sulfate removal bioreactors had high microbial diversity, but that the communities were dominated by sulfate reducers. In addition, we hypothesized that ethanol is a suitable electron donor for the biological sulfate reduction in the treatment of sulfate-rich water originating from a northern mine.

In this study, we tested the suitability of using ethanol as an electron donor for sulfate reduction for the biological treatment of sulfate-rich mine water with low metal content. The performance of the bioreactors was monitored by measuring pH, RedOx potential, and sulfide and sulfate content throughout the experiment. The microbial communities in the used laboratory-scale bioreactors were characterized using a high throughput sequencing technique and bioinformatics. We found that acetate accumulated in the bioreactors and that the sulfate removal rate was generally low when using ethanol with the tested mine water. The microbial communities in the different bioreactors consisted mostly of non-SRB, with a dominance of nitrate and nitrite reducing sulfide oxidizing taxa. A high input sulfate level and a low pH, together with undissociated H_2_S as well as the accumulation of acetate, may have inhibited growth and activity of the SRB in the bioreactors. The elevated concentration of acetate may be due to the prevalence of fermentation, which may be allowed to develop because of long HRT, and is thus a symptom rather than cause of malfunction of the bioreactors. Analysis of the microbial community gave added insight into the microbial processes taking place in the bioreactors and may serve as an indicator tool for process failure and tool for optimization of the sulfate removal process. Our study also indicated that parameters normally used for monitoring a SBR process such as redox and pH, may not be sufficient for assuring a functioning process.

## Figures and Tables

**Table 1:** Chemical parameters of the mine waste water. In addition, Ag, Be, Bi, Cd, Cr, Ph, Th, and Tl were present at <0.001 mg L^−1^, and Co, Cu, U, and V at <0.005 mg L^−1^.

Measurement	Method	Unit	Concentration
Al	ICP-MS	mg L^−1^	0.034
As	ICP-MS	mg L^−1^	0.164
B	ICP-MS	mg L^−1^	0.098
Ba	ICP-MS	mg L^−1^	0.033
Li	ICP-MS	mg L^−1^	0.288
Mo	ICP-MS	mg L^−1^	0.045
Ni	ICP-MS	mg L^−1^	0.023
P	ICP-MS	mg L^−1^	0.045
Rb	ICP-MS	mg L^−1^	0.163
Sb	ICP-MS	mg L^−1^	0.056
Se	ICP-MS	mg L^−1^	0.023
Sr	ICP-MS	mg L^−1^	1.31
Zn	ICP-MS	mg L^−1^	0.01
Ca	ICP-OES	mg L^−1^	430
Fe	ICP-OES	mg L^−1^	0.242
K	ICP-OES	mg L^−1^	132
Mg	ICP-OES	mg L^−1^	1890
Mn	ICP-OES	mg L^−1^	0.924
Na	ICP-OES	mg L^−1^	165
Si	ICP-OES	mg L^−1^	1.60
S	ICP-OES	mg L^−1^	2660
F	Metrolab internal	mg L^−1^	<0.1
Cl	Metrolab internal	mg L^−1^	26
SO_4_	Metrolab internal	mg L^−1^	8900
Ammonium-N	ISO 7150: 1984, disc. anal.	mg L^−1^	2.7
Nitrate-N	Metrolab internal	mg L^−1^	0.11
Nitrite-N	SFS 3029, autom.	mg L^−1^	1.1
Br	ISO 10304-1:2007	mg L^−1^	<1
Sb	SFS-EN ISO 17294-2:2005	µg L^−1^	58
As	SFS-EN ISO 11885:2009	µg L^−1^	150
Ni	SFS-EN ISO 17294-2:2005	µg L^−1^	15

**Table 2:** Physicochemical parameters of the effluents on day 91.

Measurement	Reactor 1		Reactor 2		Reactor 3	
Day	88 *	91	88	91	88	91
pH	6.2	7.4	n.a.	6.5	7.5	6.8
SO_4_ removal %	−1%	9	n.a.	9	9	17
Acetate mg L^−1^	2170	3467	n.a.	119	126	10
Ammonium mg L^−1^	n.a.	60	n.a.	35	n.a.	30
Phosphate mg L^−1^	n.a.	18	n.a.	13	n.a.	16
Ethanol mg L^−1^	400	50	n.a.	1220	200	10

* added 1 g NaCO_3_ to the column in order to increase pH; n.a. not analyzed.

## Abbreviations

The following abbreviations are used in this manuscript:

HRT	hydraulic retention time
SRB	Sulfate Reducing Bacteria
*dsr*B	dissimilatory sulphite reductase, subunit B
SR	Sulfate reduction
HTP sequencing	High throughput sequencing
UASB	upflow anaerobic sludge blanket reactor
FBR	fluidized bed reactors
ICP-MS	Inductively coupled plasma mass spectrometry
ICP-OES	Inductively coupled plasma optical emission spectrometry
COD	Chemical Oxygen Demand
RedOx	Reduction Oxidation
qPCR	quantitative polymerase chain reaction
OTU	Operational Taxonomic Units

## References

[1] Bowell R.J., Jarvis A.P., Dudgeon B.A., Younger P.L. (2004). A review of sulphate removal options for mine waters. “International Mine Water Association Symposium”—Mine Water 2004.

[2] Elliot P., Ragusa S., Catcheside D. (1998). Growth of sulfate-reducing bacteria under acidic conditions in an upflow anaerobic bioreactor as a treatment system for acid mine drainage. Water Res..

[3] Kaksonen A.H., Riekkola-Vanhanen M.-L., Puhakka J.A. (2003). Optimization of metal sulphide precipitation in fluidized-bed treatment of acidic wastewater. Water Res..

[4] Sahinkaya E., Gunes F.M., Ucar D., Kaksonen A.H. (2011). Sulfidogenic fluidized bed treatment of real acid mine drainage water. Bioresour. Technol..

[5] Muyzer G., Stams A.J. (2008). The ecology and biotechnology of sulphate-reducing bacteria. Nat. Rev. Microbiol..

[6] Celis L.B., Villa-Gomez D., Alpuche-Solis A.G., Ortega-Morales B.O., Razo-Flores E. (2009). Characterization of sulfate-reducing bacteria dominated surface communities during start-up of a down-flow fluidized bed reactor. Ind. Microbiol. Biotechnol..

[7] Nagpal S., Chuichulcherm S., Peeva L., Livingston A. (2000). Microbial Sulfate Reduction in a Liquid–Solid Fluidized Bed Reactor. Biotechnol. Bioeng..

[8] Cocos I.A., Zagury G.J., Clément B., Samson R. (2002). Multiple factor design for reactive mixture selection for use in reactive walls in mine drainage treatment. Water Res..

[9] Regenspurg S., Brand A., Peiffer S. (2004). Formation and stability of schwertmannite in acidic mining lakes. Geochim. Cosmochim. Acta.

[10] Baron D., Palmer C.D. (1996). Solubility of jarosite at 4–35 C. Geochim. Cosmochim. Acta.

[11] Van der Zee F.P., Villaverde S., García P.A., Fdz.-Polanco F. (2007). Sulfide removal by moderate oxygenation of anaerobic sludge environments. Bioresour. Technol..

[12] Basen M., Schut G.J., Nguyen D.M., Lipscomb G.L., Benn R.A., Prybol C.J., Vaccaro B.J., Poole F.L., Kelly R.M., Adams M.W. (2014). Single gene insertion drives bioalcohol production by a thermophilic archaeon. Proc. Natl. Acad. Sci. USA.

[13] Bertsch J., Siemund A.L., Kremp F., Müller V. (2016). A novel route for ethanol oxidation in the acetogenic bacterium Acetobacterium woodii: The acetaldehyde/ethanol dehydrogenase pathway. Environ. Microbiol..

[14] Fossing H.E., Gallardo V.A., Jorgensen B.B., Huttel M., Nielsen L.P., Schulz H., Canfield D.E., Forster S., Glud R.N., Gundersen J.K. (1995). Concentration and transport of nitrate by the mat-forming sulphur bacterium Thioploca. Nature.

[15] Greene E.A., Hubert C., Nemati M., Jenneman G.E., Voordouw G. (2003). Nitrite reductase activity of sulphate-reducing bacteria prevents their inhibition by nitrate-reducing, sulphide-oxidizing bacteria. Environ. Microbiol..

[16] Gieg L.M., Jack T.R., Foght J.M. (2011). Biological souring and mitigation in oil reservoirs. Appl. Microbiol. Biotechnol..

[17] Langille M.G., Zaneveld J., Caporaso J.G., McDonald D., Knights D., Reyes J.A., Clemente J.C., Burkepile D.E., Thurber R.L.V., Knight R. (2013). Predictive functional profiling of microbial communities using 16S rRNA marker gene sequences. Nat. Biotechnol..

[18] Muyzer G., De Waal E.C., Uitterlinden A.G. (1993). Profiling of complex microbial populations by denaturing gradient gel electrophoresis analysis of polymerase chain reaction-amplified genes coding for 16S rRNA. Appl. Environ. Microbiol..

[19] Geets J., Borremans B., Diels L., Springael D., Vangronsveld J., Van der Lelie D., Vanbroekhoven K. (2006). *Dsr*B gene-based DGGE for community and diversity surveys of sulfate-reducing bacteria. J. Microbiol. Methods.

[20] Wagner M., Roger A.J., Flax J.L., Brusseau G.A., Stahl D.A. (1998). Phylogeny of dissimilatory sulfite reductases supports an early origin of sulfate respiration. J. Bacteriol..

[21] Rajala P., Bomberg M., Vepsäläinen M., Carpén L. (2017). Microbial fouling and corrosion of carbon steel in deep anoxic alkaline groundwater. Biofouling.

[22] Bomberg M., Arnold M., Kinnunen P. (2015). Characterization of the Bacterial and Sulphate Reducing Community in the Alkaline and Constantly Cold Water of the Closed Kotalahti Mine. Minerals.

[23] Klindworth A., Pruesse E., Schweer T., Peplies J., Quast C., Horn M., Glöckner F.O. (2013). Evaluation of general 16S ribosomal RNA gene PCR primers for classical and next-generation sequencing-based diversity studies. Nucleic Acids Res..

[24] Buee M., Reich M., Murat C., Morin E., Nilsson R.H., Uroz S., Martin F. (2009). 454 Pyrosequencing analyses of forest soils reveal an unexpectedly high fungal diversity. New Phytol..

[25] Caporaso J.G., Kuczynski J., Stombaugh J., Bittinger K., Bushman F.D., Costello E.K., Fierer N., Gonzalez Pena A., Goodrich J.K., Gordon J.I. (2010). QIIME allows analysis of high-throughput community sequencing data. Nat. Methods.

[26] Edgar R.C. (2010). Search and clustering orders of magnitude faster than BLAST. Bioinformatics.

[27] DeSantis T.Z., Hugenholtz P., Larsen N., Rojas M., Brodie E.L., Keller K., Huber T., Dalevi D., Hu P., Andersen G.L. (2006). Greengenes, a chimera-checked 16S rRNA gene database and workbench compatible with ARB. Appl. Environ. Microbial..

[28] Kõljalg U., Nilsson R.H., Abarenkov K., Tedersoo L., Taylor A.F.S., Bahram M., Bates S.T., Bruns T.D., Bengtsson-Palme J., Callaghan T.M. (2013). Towards a unified paradigm for sequence-based identification of Fungi. Mol. Ecol..

[29] Wang Q., Garrity G.M., Tiedje J.M., Cole J.R. (2007). Naive Bayesian classifier for rapid assignment of rRNA sequences into the new bacterial taxonomy. Appl. Environ. Microbiol..

[30] Altschul S.F., Gish W., Miller W., Myers E.W., Lipman D.J. (1990). Basic local alignment search tool. J. Mol. Biol..

[31] R Core Team R: A Language and Environment for Statistical Computing. https://www.R-project.org/.

[32] Hammer O., Harper D.A.T., Ryan P.D. (2009). PAST-palaeontological statistics, ver. 1.89. Palaeontologia Electron..

[33] Mahmood Q., Hu B., Cai J., Zheng P., Azim M.R., Jilani G., Islam E. (2009). Isolation of *Ochrobactrum* sp. QZ2 from sulfide and nitrite treatment system. J. Hazard. Mat..

[34] Geiser D.M., Gueidan C., Miadlikowska J., Lutzoni F., Kauff F., Hofstetter V., Fraker E., Schoch C.L., Tibell L., Untereiner W.A. (2006). Eurotiomycetes: Eurotiomycetidae and chaetothyriomycetidae. Mycologia.

[35] Tsoy O., Ravcheev D., Mushegian A. (2009). Comparative genomics of ethanolamine utilization. J. Bacteriol..

[36] San Francisco B., Zhang X., Whalen K., Gerlt J. (2015). A Novel Pathway for Bacterial Ethanolamine Metabolism. FASEB J..

[37] Gibert O., De Pablo J., Cortina J.L., Ayora C. (2002). Treatment of acid mine drainage by sulphate-reducing bacteria using permeable reactive barriers: A review from laboratory to full-scale experiments. Rev. Environ. Sci. Biotechnol..

[38] Delaune R.D., Reddy K.R., Hillel D. (2005). Redox Potential. Encyclopedia of Soils in the Environment.

[39] Kaksonen A. (2004). The performance, kinetics and microbiology of sulfidogenic fluidized-bed reactors treating acidic metal- and sulfate-containing wastewater. Ph.D. Dissertation.

[40] Moosa S., Harrison S.T.L. (2006). Product inhibition by sulfide species on biological sulphate reduction for the treatment of acid mine drainage. Hydrometallurgy.

[41] White C., Gadd G.M. (1996). Mixed sulphate-reducing bacterial cultures for bioprecipitation of toxic metals, factorial and response-surface analysis of the effects of dilution rate, sulphate and substrate concentration. Microbiology.

[42] Oyekola O.O., Van Hille R.P., Harrison S.T.L. (2010). Kinetic analysis of biological sulphate reduction using lactate as carbon source and electron donor: Effect of sulphate concentration. Chem. Eng. Sci..

[43] Dar S.A., Kleerebezem R., Stams A.J., Kuenen J.G., Muyzer G. (2008). Competition and coexistence of sulfate-reducing bacteria, acetogens and methanogens in a lab-scale anaerobic bioreactor as affected by changing substrate to sulfate ratio. Appl. Microbiol. Biotechnol..

[44] Kristjansson J.K., Schönheit P. (1983). Why do sulfate-reducing bacteria outcompete methanogenic bacteria for substrates?. Oecologia.

[45] Ozuolmez D., Na H., Lever M.A., Kjeldsen K.U., Jørgensen B.B., Plugge C.M. (2015). Methanogenic archaea and sulfate reducing bacteria co-cultured on acetate: Teamwork or coexistence?. Front. Microbiol..

